# Energy Sprawl or Energy Efficiency: Climate Policy Impacts on Natural Habitat for the United States of America

**DOI:** 10.1371/journal.pone.0006802

**Published:** 2009-08-26

**Authors:** Robert I. McDonald, Joseph Fargione, Joe Kiesecker, William M. Miller, Jimmie Powell

**Affiliations:** 1 Worldwide Office, The Nature Conservancy, Arlington, Virginia, United States of America; 2 Central Region, The Nature Conservancy, Minneapolis, Minnesota, United States of America; 3 Rocky Mountain Region, The Nature Conservancy, Fort Collins, Colorado, United States of America; 4 Department of Chemical and Biological Engineering, Northwestern University, Evanston, Illinois, United States of America; 5 Worldwide Office, The Nature Conservancy, Arlington, Virginia, United States of America; Universidade de Vigo, Spain

## Abstract

Concern over climate change has led the U.S. to consider a cap-and-trade system to regulate emissions. Here we illustrate the land-use impact to U.S. habitat types of new energy development resulting from different U.S. energy policies. We estimated the total new land area needed by 2030 to produce energy, under current law and under various cap-and-trade policies, and then partitioned the area impacted among habitat types with geospatial data on the feasibility of production. The land-use intensity of different energy production techniques varies over three orders of magnitude, from 1.9–2.8 km^2^/TW hr/yr for nuclear power to 788–1000 km^2^/TW hr/yr for biodiesel from soy. In all scenarios, temperate deciduous forests and temperate grasslands will be most impacted by future energy development, although the magnitude of impact by wind, biomass, and coal to different habitat types is policy-specific. Regardless of the existence or structure of a cap-and-trade bill, at least 206,000 km^2^ will be impacted without substantial increases in energy efficiency, which saves at least 7.6 km^2^ per TW hr of electricity conserved annually and 27.5 km^2^ per TW hr of liquid fuels conserved annually. Climate policy that reduces carbon dioxide emissions may increase the areal impact of energy, although the magnitude of this potential side effect may be substantially mitigated by increases in energy efficiency. The possibility of widespread energy sprawl increases the need for energy conservation, appropriate siting, sustainable production practices, and compensatory mitigation offsets.

## Introduction

Climate change is now acknowledged as a potential threat to biodiversity and human well-being, and many countries are seeking to reduce their emissions by shifting from fossil fuels to other energy sources. One potential side effect with this switch is the increase in area required by some renewable energy production techniques [1]–[5]. Energy production techniques vary in the spatial extent in which production activities occur, which we refer to as their energy sprawl [2], [3], defined as the product of the total quantity of energy produced annually (e.g., TW hr/yr) and the land-use intensity of production (e.g. km^2^of habitat per TW hr/yr). While many studies have quantified the likely effect of climate change on the Earth's biodiversity due to climate-driven habitat loss, concluding that a large proportion of species could be driven extinct [6]–[8], relatively few studies have evaluated the habitat impact of future energy sprawl. It is important to understand the potential habitat effects of energy sprawl, especially in reference to the loss of specific habitat types, since habitats vary markedly in the species and ecosystem processes they support.

**Figure 1:**
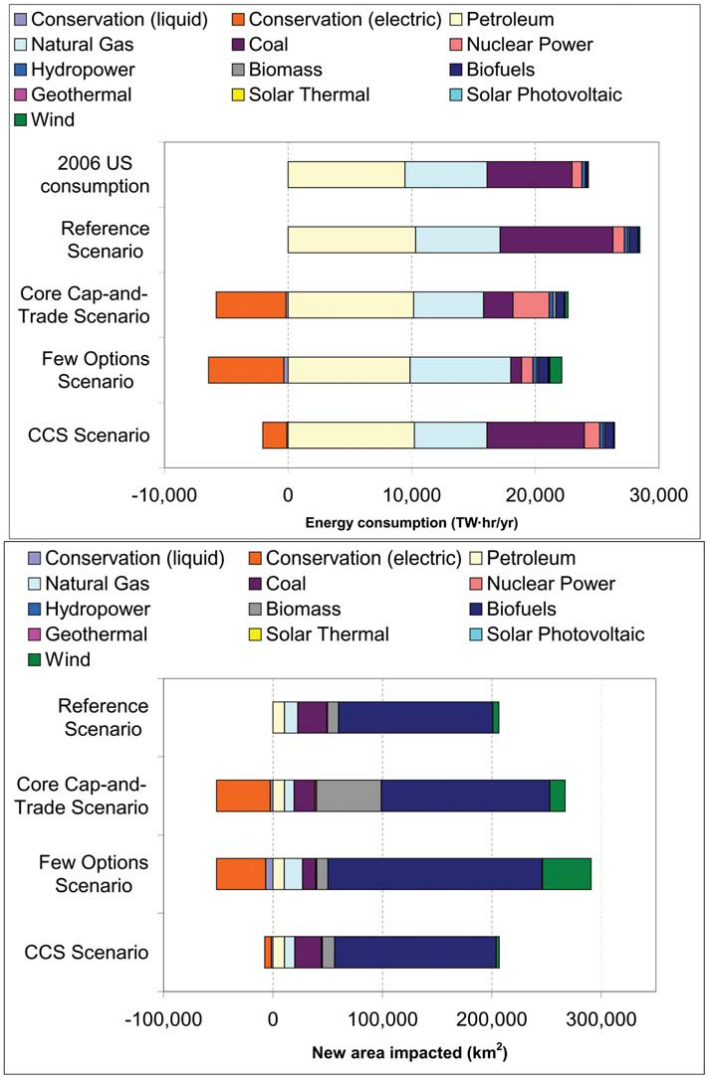
U.S. energy consumption and total new area impacted. (A) U.S. energy consumption in 2006 and under four EIA scenarios. Energy conservation of liquid fuels and electricity, calculated relative to the Reference scenario, are shown as negative since they reduce consumption. (B) The total new area impacted because of development between 2006 and 2030. The new area impacted, or energy sprawl, is a product of consumption and the land-use intensity values in Figure 3. Energy conservation is calculated based on a scenario-specific weighted-average of the energy mix.

Within the United States, the world's largest cumulative polluter of greenhouse gases, concern over climate change has led to the consideration of a cap-and-trade system to regulate emissions, such as the previously proposed Lieberman-Warner Climate Security Act (S. 2191) [9] and the Low Carbon Economy Act (S. 1766) [10]. Major points of contention in structuring a cap-and-trade system are the feasibility and desirability of carbon capture and storage (CCS) at coal plants, the creation of new nuclear plants, and whether to allow international offset programs that permit U.S. companies to meet obligations abroad [11]. The rules of a cap-and-trade system, as well as technological advances in energy production and changes in the price of fossil fuels, will affect how the U.S. generates energy. In this study we take scenarios of a cap-and-trade system's effect on United States energy production and evaluate each scenario's impact on habitat due to energy sprawl. Our scenarios (Fig. 1A) are based on the Energy Information Administration (EIA) forecast of energy production in 2030 [12] under current law (the “Reference Scenario”), including the renewable fuel standard of the Energy Independence and Security Act of 2007, and under three cap-and-trade scenarios: the “Core Cap-and-Trade Scenario”, where the full Lieberman-Warner Climate Change Act is implemented; the “Few Options Scenario”, where international offsets are not allowed and where new nuclear production and coal production with CCS are not possible; and the “CCS Scenario”, where Congress enacts the Low Carbon Economy Act, a cap-and-trade system more favorable to coal with CCS.

**Figure 2:**
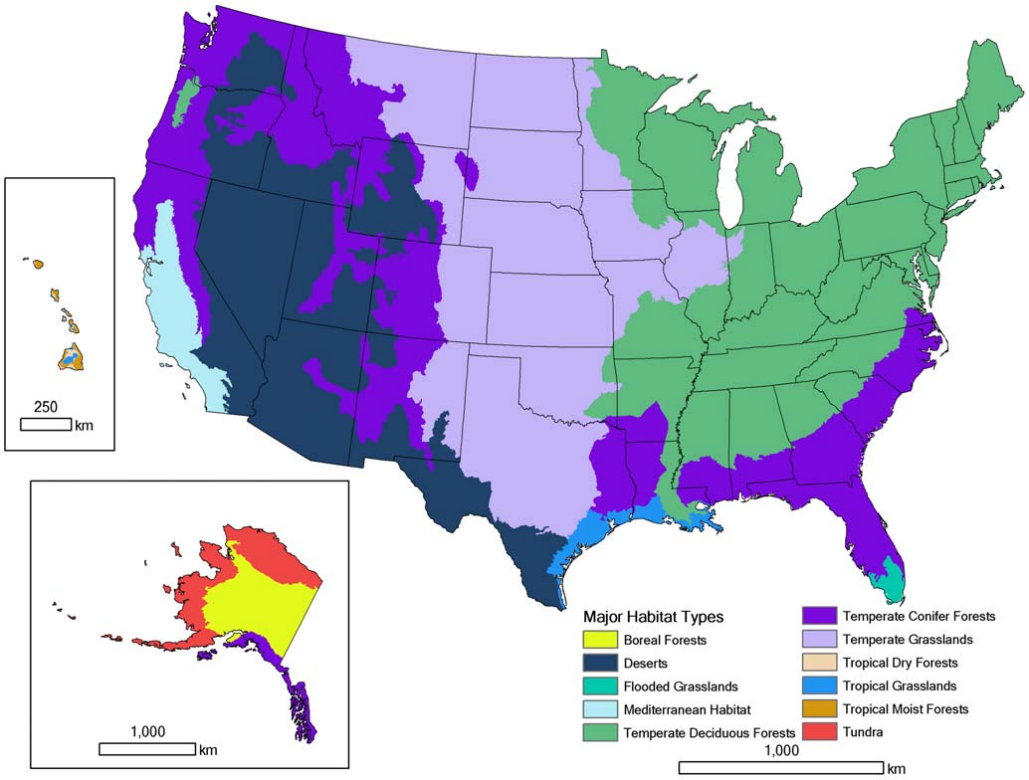
Major habitat types used to analyze the land-use implications of EIA scenarios. Within each major habitat type, there are a variety of land-uses, from relatively wild places to agricultural and urban systems. Our analysis estimates the new area needed for energy development within each major habitat type, without specifying where within each major habitat type this energy development might occur.

Under each scenario, we first estimate the total new land area in the U.S. needed to produce energy for each production technique as a function of the amount of energy needed and the land-use intensity of production. We examine the effect of U.S. climate policy on future energy sprawl using energy scenarios based on proposed legislation, building on a body of literature on this topic [1], [2], [13]–[15]. Note that our analysis focuses only on U.S. land-use implications, ignoring other, potentially significant international land-use implications of U.S. climate policy. Second, we use available information on where new energy production facilities would be located to partition this area among major habitat types (Fig. 2). We calculate the new area directly impacted by energy development within each major habitat type, but do not attempt to predict where within each major habitat type energy development will take place, nor possible indirect effects on land-use regionally or globally due to altered land markets. Our analysis provides a broad overview of what change in the energy sector will mean for area impacted in different natural habitat types, recognizing that such a broad analysis will inevitably have to simplify parts of a complex world.

## Results

### Land-use intensity of energy production

**Figure 3:**
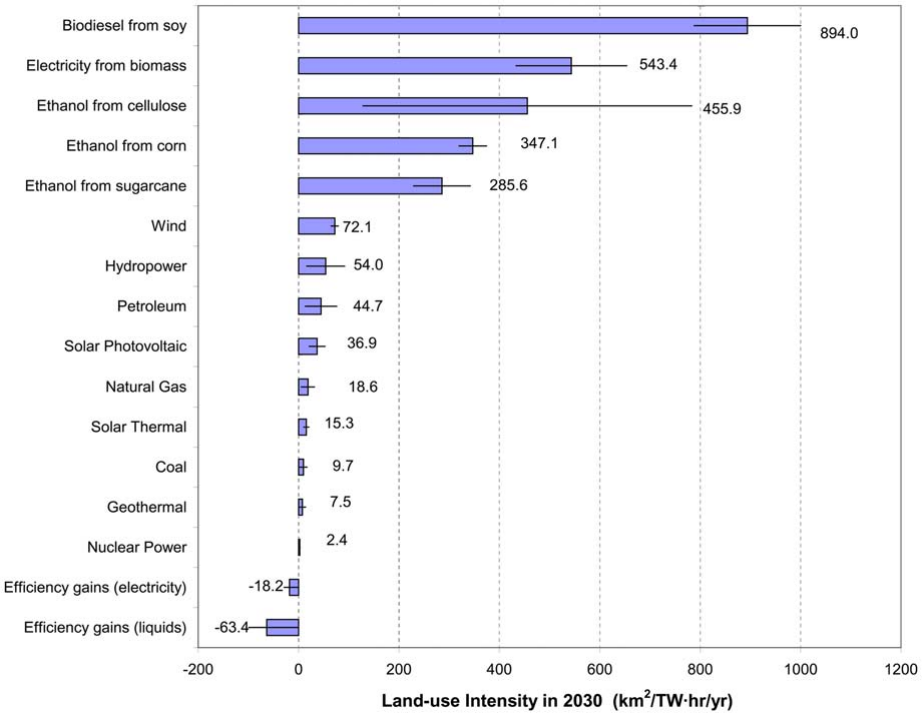
Land-use intensity for energy production/conservation techniques. Value shown is for 2030, as measured in km^2^ of impacted area in 2030 per terawatt-hour produced/conserved in that year. Error bars show the most-compact and least-compact estimates of plausible current and future levels of land-use intensity. Numbers provided are the midpoint between the high and low estimates for different techniques. For liquid fuels, energy loss from internal combustion engines is not included in this calculation.

The land-use intensity of different energy production techniques (i.e., the inverse of power density [16], [17]), as measured in km^2^ of impacted land in 2030 per terawatt-hour per year, varies over three orders of magnitude (Fig. 3). Nuclear power (1.9–2.8 km^2^/TW hr/yr), coal (2.5–17.0 km^2^/TW hr/yr) and geothermal (1.0–13.9 km^2^/TW hr/yr) are the most compact by this metric. Conversely, biofuels (e.g., for corn ethanol 320–375 km^2^/TW hr/yr) and biomass burning of energy crops for electricity (433–654 km^2^/TW hr/yr) take the most space per unit power. Most renewable energy production techniques, like wind and solar power, have intermediate values of this metric.

Energy conservation can reduce overall energy consumption thus reducing the area impacted by energy development. For every TW hr decrease in annual electric power consumption, a weighted-average of electricity use under the Reference scenario suggests 7.6–28.7 km^2^ of avoided impact. The corresponding figure for liquid fuels (27.5–99.3 km^2^ of avoided impact per TW hr/yr) is higher because of the relatively large land-use intensity of biofuels.

Our definition of impact varies among energy production techniques, so a less compact way of generating energy does not necessarily mean that an energy production technique is more damaging to biodiversity, but simply that it has a larger spatial area impacted to some degree. Moreover, many energy production techniques actually have multiple effects on biodiversity, which operate at different spatial and temporal scales. Biodiversity impacts that are likely to scale with areal impact include habitat replacement and habitat fragmentation. Energy production impacts on biodiversity not related to land use intensity include impacts on air quality (e.g. acid rain, particulates), water quality (e.g. mercury, eutrophication), water consumption (e.g. irrigation water, evaporation from hydroelectric reservoirs), and water flows (e.g. dam-based hydroelectric). Further, the longevity of the impacts described here varies. For example, radioactive nuclear waste will last for millennia, some mine tailings will be toxic for centuries, and other mines may be reclaimed for agriculture within decades.

A full discussion of the impacts on biodiversity of energy production is beyond the scope of this paper, but one fundamental distinction is worth making. Some energy production techniques clear essentially all natural habitat within their area of impact. A review of the literature (see citations below and in Supplementary Text S1) found this to be true for coal, nuclear, solar, and hydropower, as well as for the growth of energy crops for biofuels or for burning for electricity. Energy crop production is a particularly complex situation because even if new energy crop production occurs on land that was previously in agricultural production, remaining global demand for agricultural commodities may spur indirect effects on land-use elsewhere, potentially causing an agricultural expansion in areas far from the location of energy crop production [18]. Other energy production techniques have a relatively small infrastructure footprint and a larger area impacted by habitat fragmentation and other secondary effects on wildlife. A review of the literature found that production techniques that involve wells like geothermal, natural gas, and petroleum have about 5% of their impact area affected by direct clearing while 95% of their impact area is from fragmenting habitats and species avoidance behavior. Wind turbines have a similar figure of about 3–5% of their impact area affected by direct clearing while 95–97% of their impact area is from fragmenting habitats, species avoidance behavior, and issues of bird and bat mortality.

### Energy sprawl in 2030

Regardless of climate change policy, the total new area affected by energy production techniques by 2030 exceeds 206,000 km^2^ in all scenarios (Fig. 1B), an area larger than the state of Nebraska. Biofuels have the greatest cumulative areal impact of any energy production technique, despite providing less than 5% of the U.S. total energy under all scenarios. Biofuel production, and hence new area impacted, is similar among scenarios because EIA's economic model suggests that, under current law, incentives for biofuel production cause expansion of this energy production technique regardless of climate policy.

**Figure 4:**
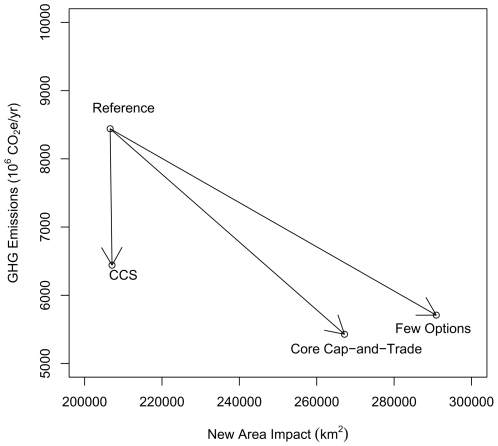
Greenhouse gas emissions and total new area impacted with a cap-and-trade system. Arrows depict the difference between the Reference Scenario, with no cap-and-trade system, and three other scenarios where a cap-and-trade system is implemented. Greenhouse gas emissions measured in million tonnes of carbon dioxide equivalent.

Nevertheless, in the scenarios we considered there is a tendency for greater reductions in greenhouse gas emissions to be associated with a greater total new area affected by energy development, particularly under the Core Cap-and-Trade and Few Options Scenario (Figure 4). A decrease in U.S. emissions increases the new area impacted, although the magnitude of the effect is policy specific. Under the Core Cap-and-Trade scenario, the burning of energy crops for electricity becomes profitable after the price of electricity rises due to the cap-and-trade system, resulting in a large new areal impact. Similarly, wind power is very important in the Few Options scenario, where new electric production from coal and nuclear is not an option, and causes a large new areal impact. Conversely, in scenarios where there is not control on carbon emissions (Reference Scenario) or in cases where CCS is viable (e.g., CCS Scenario), coal production has a large new areal impact. The infrastructure for CCS is actually a small fraction of the area impacted by coal mining itself, so the major land-use change implication of the viability of coal with CCS is the continuation of coal mining (Supplementary Data S1).

Our results stress the importance of energy conservation for reducing energy sprawl. Relative to the Reference scenario, all cap-and-trade scenarios involve a reduction in energy consumed (Fig. 1B), because of energy efficiency and foregone consumption due to higher energy prices. This energy conservation is primarily in the electricity market, which is more elastic than demand for liquid fuels. Electricity conservation avoids impacts on at least 49,600 km^2^ in the Core Cap-and-Trade scenario, while at least 2,500 km^2^ will be saved due to liquid fuel conservation, compared to the Reference scenario. EIA assumptions about the potential for energy conservation are relatively modest [19] and some groups argue that energy conservation has greater potential [20].

### Habitat impacts

The major terrestrial habitat types (Fig. 2) impacted domestically by energy development varied among energy production technique (Table 1). Regardless of scenario, the major habitat types with the most new area affected, summing over all energy production techniques, are Temperate Deciduous Forests and Temperate Grasslands (Supplementary Data S2). In the Reference scenario, Temperate Deciduous Forests have between 95,000 km^2^ (most compact estimate) and 229,000 km^2^ (least compact estimate) impacted, while Temperate Grasslands have 65,000–168,000 km^2^ impacted. In the Core Cap-and-Trade scenario these types have 119,000–254,000 km^2^ and 88,000–191,000 km^2^ impacted, respectively. Patterns of total new areal impacts are driven by biofuel production, which peaks in these two habitat types. Biomass burning for electricity and coal mining are also concentrated in Temperate Deciduous Forests and Temperate Grasslands. Wind production onshore is likely to affect Temperate Conifer Forests and Temperate Grasslands in the western U.S. disproportionately. The least impacted habitats are: Tundra; Boreal Forest; Tropical Dry Forests; Flooded Grasslands; and Tropical Moist Forests. All of these habitat types have less than 150 km^2^ impacted by energy development in the Reference Scenario and less than 600 km^2^ impacted by energy development in the Core Cap-and-Trade Scenario, using the minimal sprawl estimates from Figure 3.

**Table 1:** Minimum new area (km^2^) of habitat types impacted in the U.S.

Habitat Type	Coal	Biomass	Biofuels	Wind
Boreal Forests	94 (−27)	2 (+12)	3 (+0)	6 (+9)
Deserts	2,310 (−662)	257 (+1244)	372 (+14)	884 (+1,300)
Flooded Grasslands	0 (0)	30 (+143)	41 (+1)	0 (0)
Mediterranean Habitat	5 (−1)	123 (+596)	1,699 (+37)	54 (+79)
Temperate Conifer Forests	4,936 (−1,413)	1,883 (+9,106)	12,977 (+739)	2,835 (+4,169)
Temperate Deciduous Forests	10,297 (−2,945)	4,014 (+19,415)	76,841 (+6,751)	428 (+630)
Temperate Grasslands	7,508 (−2,147)	3,760 (+18,185)	46,821 (+4,136)	1,392 (+2,047)
Tropical Dry Forests	0 (0)	4 (+18)	5 (0)	34 (+50)
Tropical Grasslands	1,304 (−373)	59 (+284)	1,583 (+65)	3 (+5)
Tropical Moist Forests	0 (0)	7 (+32)	9 (0)	78 (+115)
Tundra	0 (0)	0 (0)	0 (0)	0 (0)

Data are from the Reference Scenario for four major types of energy development: coal production, biomass burning for electricity, biofuel production, and wind power. Energy development is partitioned among habitat types, as depicted in Figure 2. Numbers in parentheses are the change in value under the Core Cap-and-Trade Scenario. For example, boreal forests have 94 km^2^ affected under the Reference Scenario by coal, but have 27 fewer km^2^ affected by coal under the Core Cap-and-Trade Scenario (i.e., 67 km^2^).

The major habitats impacted by new energy development also varied among scenarios for certain energy production techniques. For example, scenarios where continued coal power generation is viable, either because of no restrictions on carbon emissions (Reference scenario) or because CCS is viable (e.g., CCS scenario) have greater impacts on those major habitat types with major coal seams (e.g., Temperate Deciduous Forests). Conversely, scenarios where coal power generation is less viable have greater production from wind, affecting specific habitat types where those production techniques are favorable (e.g., Temperate Conifer Forests). Climate policy thus controls the extent to which specific habitat types are at risk from new energy development.

## Discussion

Our analyses show that, regardless of scenario, at least 206,000 km2 of new land will be required to meet U.S. energy demand by 2030. Further, implementing a cap-and-trade system may increase the total new area impacted by energy development and change its distribution among habitat types, relative to the Reference scenario. Energy production will shift from fossil fuels to energy production techniques that draw more diffuse energy from a broader spatial area. Note that because the EIA analysis assumes that the energy market responds to price signals and does not explicitly attempt to minimize land-use *per se*, it is theoretically possible that there are other, more expensive mixes of energy production that would satisfy U.S. energy needs in 2030 but would take less space. Although policies that reduce carbon emissions with minimal new land use are possible, none of the different policy EIA scenarios we considered were designed with that goal in mind. As shown by Wise et al. [21], if there were financial incentives to minimize land-use in energy production like a tax on greenhouse gas emissions from land-use change, the energy market response to a cap-and-trade might be very different from the response depicted in the EIA scenarios.

There are at least four ways to achieve emissions reduction but avoid the potential side effect of energy sprawl. First, energy conservation can help reduce the new energy needed by the U.S., reducing the area impacted by new energy development. Second, because end-use generation of electricity often occurs on already developed sites, it has minimal habitat impacts, and policy instruments that encourage end-use generation can also decrease the total area impacted. Third, our results suggest that energy sprawl is less severe when the cap-and-trade bill is more flexible, allowing for CCS, new nuclear plants, and international offsets. Fourth, many areal impacts can be mitigated or eliminated with appropriate site selection and planning for energy development. The new area affected by energy development within each major habitat type might, for example, have minimal biodiversity effects if sited in already disturbed places.

The areal impacts on habitat types will vary among scenarios, along with the potential biodiversity impacts of U.S. climate policy. While not all impacts on biodiversity are strictly related to the areal impact, it is likely that energy production techniques with a large areal impact will have a relatively large biodiversity impact. Thus, the details of climate change policy, by favoring particular energy production techniques, pick biodiversity winners and losers. For instance, the Few Options Scenario assumes that international offsets, actions taken abroad to prevent carbon emissions or sequester more carbon, are not allowed under a cap-and-trade regime. The major response forecasted by EIA is an increase in wind production domestically relative to the Reference Scenario, affecting especially Temperate Conifer Forests and Temperate Grasslands. This increase in wind production may be compatible with biodiversity if properly sited, but certainly will pose a challenge for conservationists, because of the large area impacted and the threat of bird and bat mortality [22]. On the other hand, the biodiversity impacts of international offsets are beyond the scope of this paper, but could conceivably be negligible (e.g., scrubber on Chinese coal plant smokestacks), negative (e.g., replacing natural grasslands with plantation forests), or positive (e.g., reducing emissions from degradation or deforestation).

Regardless of whether or not a cap-and-trade system is implemented, the EIA analysis forecasts that biofuels will increase dramatically in importance, with large areal impacts. In the Reference scenario 141,000–247,000 km^2^ will be impacted by biofuels. Within the United States much energy crop production will occur on grassland or forest sites already in use for agricultural production, although increased aggregate demand for agricultural commodities may still spur agricultural expansion domestically or internationally (i.e., indirect land-use effects). In part, the large increase in biofuels forecasted by EIA simply reflects their assumption that current law, including the renewable fuel standard defined by the Energy Independence and Security Act of 2007, is maintained to 2030. In part it also reflects the inelasticity of the market for liquid fuels, relative to the electricity market, and the likely high cost of petroleum over the long-term [23]. It seems likely that under current law there will be a large areal impact from biofuels regardless of the cap-and-trade system put in place. Given the high land-use intensity of biofuels, techniques for either increasing the efficiency of biofuel production or making sites of energy crop production more biodiversity-friendly should be a high priority for research.

Our results demonstrate that, under certain policy scenarios, one potential side effect of reducing emissions is an increase in habitat impacts. The impact of a cap-and-trade system will be less, however, than from biofuel production already mandated by current law. Aggressive energy conservation, appropriate siting, sustainable production practices, and reduction of greenhouse gas emissions will all be necessary to minimize the impact of future energy use on habitat and wildlife. Energy sprawl deserves to be one of the metrics by which energy production is assessed.

## Materials and Methods

Our analysis proceeded in two phases. First, we calculated the new land area of energy development necessary to meet the EIA scenarios. Not all biodiversity impacts are directly related to the amount of land taken up by a technology, but it is likely that an energy production technique that takes a lot of land will have a relatively large biodiversity impact, so the total new area impact is a useful quantity to measure. Second, we partitioned this new land area among different geographic regions. We focused on domestic impacts for this analysis, ignoring the future habitat impacts of foreign-produced energy, principally future oil imports from Canada, Latin America, and the Middle East, as well as future ethanol imports from Brazilian sugarcane plantations. Similarly, we focused on terrestrial impacts for this analysis, ignoring potential freshwater or marine impacts by hydropower, wind, oil, and natural gas development in U.S. waters. Finally, we are calculating direct land-use (how much land will we need to produce energy?), and did not attempt to estimate secondary changes in the land market in response to the direct land-use.

### Scenarios

Our analysis is based on the EIA's 2008 scenarios of energy markets and the economy [12]. These scenarios were calculated by EIA's National Energy Modeling System, a comprehensive econometric model of U.S. energy production, imports, and consumption. The scenarios supply information on the amount of additional energy consumed in 2030 in a particular sector (e.g., million barrels per day of petroleum, billion KW hr of new generation capacity by solar power). We use four scenarios in our analysis. A Reference scenario describes what will likely happen in U.S. energy markets under laws in force as of April 2008. The Core Cap-and-Trade scenario forecasts the effect of the full Lieberman-Warner Climate Security Act (S. 2191), which regulates emissions of greenhouse gases through a cap-and-trade system and provides economic incentives for increased energy efficiency [24]. One variant of S. 2191 is considered, the Few Options scenario, where the use of international offsets in greenhouse gas emissions is either not economically feasible or is severely limited by regulation and where there is no increase in nuclear, coal with CCS, and imports of liquefied natural gas over current levels (the EIA called this the “Limited Alternatives/No International Offsets case”). Finally, the CCS scenario forecasts the effect of the Low Carbon Economy Act (S. 1766), which also sets up a cap-and-trade system for greenhouse gases but offers strong incentives for the development and deployment of CCS [25]. Overall energy consumption by each sector for each scenario is shown in Figure 1A.

Four things about the scenarios are worth noting. First, other scenarios of the likely effect of S. 2191 [11], [24] are available from other groups, although they are broadly similar to the EIA analysis. Second, all scenarios of the effect of a cap-and-trade bill are tentative due to uncertainty about the pace of technological change, among other things. Thus, the EIA scenarios we use in this analysis must be taken as indicative of future trends, but not definitive [24]. Third, the EIA scenarios model the likely response of the U.S. energy sector in response to a set of policy assumptions, and do not consider land-use *per se*. The EIA scenarios predict the most likely response, and do not attempt to find a more expensive energy mix that would minimize the total land-use. Finally, U.S. energy policy has changed rapidly since the EIA's 2008 analysis, which still is the most current available full analysis of a cap-and-trade bill. The Warner-Lieberman bill is no longer considered an active bill, and most activity in Congress has focused on the Waxman-Markey bill (H.R. 2454), which proposes a very similar cap-and-trade system. Additionally, the passage of the American Recovery and Reinvestment Bill of 2009 (i.e., the stimulus bill) has provided significant support to renewable energy producers, particularly wind producers. Thus, the Reference Case discussed in this manuscript may underestimate the amount of renewable energy production in that case. Despite these changes in U.S. climate policy since the EIA's 2008 analysis, the broad results of our analysis will likely apply to any similar cap-and-trade system.

### Calculating area requirements

Our general strategy was to estimate reasonable most-compact and least-compact values of the amount of area needed to produce a certain amount of energy in a year, the land-use intensity of production. We then multiplied the needed energy by our measurement of land-use intensity of production (km^2^/energy/year) to obtain the “energy sprawl,” the total new area needed for new energy production in that sector. Calculation of land-use intensity in this manner is useful for the goals of our analysis, allowing calculation of the total new area impacted by energy development. It is similar to the measurement of “area efficiency” [26], in that it does not attempt to account for site preparation prior to energy production nor potential site reclamation after production has ceased, which are sometimes considered in a full life-cycle analysis [3]. Ecologically, over the time period of our study few sites will be reclaimed to vegetation approaching their original habitat value [27]. Numerically, our land-use intensity values represent a lower estimate because they do not include land-use during site preparation or reclamation. While our methodology does not allow a full statistical analysis of the uncertainty of our estimates, the variation between the least compact and most compact estimates of land-use intensity captures most of the uncertainty.

Estimating areal impacts of new methods of electricity generation is relatively straightforward. The EIA forecasts new power generating capacity needed (billion KW), after accounting for likely retirement of existing generation capacity and the nameplate capacity factors of different energy production techniques. We used values from the literature to calculate the km^2^ of impact per GW of new generating capacity (see Tables 1, 2, and Supplementary Text S1). Our approach ignores the importation of electricity from Canada or Mexico, on the grounds that this is predicted by the EIA to remain a minor component of total electricity generation. End-use generation of electricity, which is tracked separately for the EIA for several sectors, is considered to have negligible area requirements, since it by definition occurs on previously developed sites. Similarly, the energy efficiency increases in the EIA scenarios are considered to have negligible area requirements, since they occur through upgrades in existing building and infrastructure. We have not attempted to estimate the new area impacted by new long-distance transmission lines needed to carry new capacity, as the length and location of new lines is very uncertain and depends on future energy production mixes as well as federal and state policy.

**Table 2:** Land-use intensity of production for coal mining.

Geographic region	Proportion surface mining	Most compact ha/mmt	Least Compact ha/mmt	Notes
Appalachia U.S.	0.352	11.6 pit, 79.1 surface	115.7 pit, 791.4 surface	Proportion of surface mining from EIA's 2006 Coal production in the United states fact sheet (uses 2003 data). Surface coal yields most compact figure based on Spitzley and Keoleian [3], least compact figure on Flattop mine has 2.3 million tons of coal on 600 acres. Pit mining assumed 10% of area as surface mining.
Interior U.S.	0.643	11.6 pit, 79.1 surface	115.7 pit, 791.4 surface	As above
Western U.S.	0.898	11.6 pit, 79.1 surface	115.7 pit, 791.4 surface	As above
Import	0.671	11.6 pit, 79.1 surface	115.7 pit, 791.4 surface	Proportion surface mining assumed to be same as overall U.S. average.

For each geographic region of coal, we show the proportion of the coal that is from surface mines versus pit mines, as well as least-compact and most-compact estimates of the area requirements of coal mining, in hectares per million metric tonnes of coal. Impact for coal mines is defined as the area directly surrounding the mine site.

Our estimates of land-use intensity of production are shown in Table S1 and Supplementary Text S1. Our large-area estimates are generally from current operating plants, whereas our small-area estimates represent expert opinion about expected future technological advances in efficiency. The definition of impact implied by these estimates was designed to match the majority of published studies of the severity of impact on biodiversity, and thus varies slightly among energy technologies. For example, for hydropower we have assumed that new dam construction inundates an area of terrestrial habitat, removing it of most of its native biodiversity. Note also that the different categories of electricity generation derive from the EIA report. For instance, the EIA chose to recognize solar photovoltaic and electricity from solar thermal as two different energy production techniques, and we follow their convention in our analysis. The EIA partitioned solar photovoltaic use between end-users, assumed to have negligible land-use implications in our analysis, and large-scale generation, which does have significant areal implications. We track end-use and large-scale generation separately in this and other cases, following EIA's methodology.

The EIA forecasts biofuel (ethanol, biodiesel, and other liquids from biomass) production and total use, with the vast majority of use being ethanol in transportation. Moreover, they estimate the proportion of domestic ethanol production from corn, cellulose, and other feedstocks, as well as net imports of ethanol and biodiesel. For each type of biofuel and its feedstock, we estimated least compact and most compact estimates of the number of m^2^ of feedstock cropland per liter of biofuel (Table S2 and Supplementary Text S1). In general, least compact estimates are for current agricultural yields (kg/m^2^) and biofuel production efficiencies (L/kg), while most compact estimates are a product of future agricultural yields and biofuel production efficiencies. For some crops like soy, the difference in least compact and most compact estimates is primarily due to a predicted increase in yield, while for other biofuels like cellulosic ethanol the difference is largely due to differences in biofuel production efficiency.

For many biofuels, farmers also make a portion of their income from coproducts, portions of the crop that are not used to make biofuels but have another economic market. We use a market-value allocation approach, defining the actual increase in production area of a crop as a function of the fraction of the economic value of the crop that is embodied in the biofuel [28]. Note that our methodology tracks the direct land-use needs of biofuel production, and does not consider indirect effects on land-use via agricultural commodity markets. For example, if a soy field in the U.S. is switched to corn to make ethanol, than soy production will likely expand elsewhere either domestically or internationally. A full accounting of the demand and supply curves of the various agricultural crops, biofuels, and their coproducts is beyond the scope of this project, but is an active area of research [18], [29]-[31].

Estimating the areal impact of fossil fuels was done in an analogous manner. EIA analyses divided domestic coal production into three geographic regions (Appalachia, Interior, and West). We separated the coal produced in each region into the proportion mined underground and the proportion mined at the surface, using EIA's factsheet on coal production in the United States. Then, using data on the amount of coal removed per unit area, we calculated area impacted (Table 2). For this analysis, we ignore the relatively small areal impact of coal burning power plants, which comprise a small fraction of the areal impact of coal mining.

For oil production, the EIA estimated imports as well as domestic production from three geographic regions: the land surface of the contiguous 48 United States (lower 48 onshore); water bodies in or close to the contiguous 48 United States (lower 48 offshore) and the state of Alaska. Note that because EIA assumes existing law will continue, including the ban on new oil drilling in the Arctic National Wildlife Refuge, Alaska oil production actually falls slightly under all scenarios. Based on historical data from the United States Geological Survey (USGS), we estimated the proportion of oil production that is from oil wells (as opposed to incidental production from gas wells) and the average number of barrels per day per development well (Table 3). We also estimated the number of development wells that are abandoned per year. Using these data, we calculated the number of new development wells needed to maintain current production and the number of wells needed to achieve any production increase forecasted by the EIA. By using this approach we are accounting for the tendency of older wells to fall in production over time and be abandoned, necessitating new wells just to maintain current production levels.

**Table 3:** Land-use intensity of oil and natural gas production.

Natural Resource	Proportion from well type	Average production	Most compact (ha/well)	Least Compact (ha/well)	Notes
Oil	0.862 from oil wells for onshore production in lower 48, 0.636 for Alaska.	1.14 m^3^/day of crude from lower 48 onshore, 56.13 m^3^/day of crude from Alaska onshore.	5.67	32.38	See text for estimation of trends in oil production. Pinedale Anticline spacing is taken as most-compact estimate and Jonah field spacing as least-compact estimate.
Natural Gas	0.948 from natural gas wells for U.S. onshore.	2,821 m^3^/day of dry natural gas from U.S. onshore.	5.67	32.38	As above.

For each geographic region (lower 48 onshore, lower 48 offshore, and Alaska), we used estimates of the proportion of the resource that is withdrawn from each type of well and average well productivity, as well as least-compact and most-compact estimates of the area affected by each well, in hectares per well. Impact for wells is defined as both the well area and the surrounding habitat fragmented by wells, access roads, and other structures. See text for details on impact calculations of oil and gas pipelines.

For natural gas production the EIA provides one aggregate domestic production figure, but does differentiate between pipeline natural gas imports, which are predicted to decline, and liquefied natural gas imports, which are predicted to increase. Following a similar approach to the petroleum case, we estimated the proportion of gas production that is from gas wells (as opposed to incidental production from oil wells) and the average annual thousand cubic feet per well (Table 3). Methodology generally followed that used for oil wells.

The EIA provides explicit estimates of the emissions avoided (million metric tons of CO_2_ equivalent) by the use of CCS technology, relative to a 2006 baseline, for three sectors of electricity generation (petroleum, natural gas, and coal). Because of the EIA assumptions about the cost-effectiveness of implementation of CCS and the incentive structures in S. 2191 and S. 1766, power plants burning petroleum for electricity do not generally implement CCS, whereas power plants burning natural gas or coal do whenever there is a carbon cap in place (i.e., not the Reference scenario) and when the CCS technology is available (i.e., not the Few Options case).

For new petroleum and natural gas production, we estimated the amount of new pipeline needed, based on current ratios of kilometers of pipeline to wells, assuming that these ratios held constant into the future (0.9 km/well for oil production, 1.3 km/well for gas production). For CCS, we also estimated the new pipelines needed to move CCS (0.5 km/well): the length of new pipeline per CCS injection site is likely to be more limited than in the petroleum or natural gas case because CO_2_ has little economic value [32]. For all pipelines, we then estimated the area impacted on either side of the pipe (most-compact estimate 0.3 ha/km of pipe, least-compact estimate 1.8 ha/km of pipe, based on common right of ways of pipelines). By estimating the area impacted by pipelines in this way, we are assuming that the process of pipeline construction removes most native biodiversity, and that any revegetation after pipeline construction will have minimal biodiversity value.

A literature review revealed that many energy production techniques actually have multiple effects on biodiversity, which operate at different spatial and temporal scales. A full discussion of the impacts on biodiversity of energy production is beyond the scope of this paper, but we recorded quantitative data on the proportion of our defined impact zone that was directly affected by land clearing, as opposed to more diffuse processes such as habitat fragmentation and organism avoidance behavior. Studies with useful quantitative or semi-quantitative data on this topic include: Coal [33], Nuclear [34]–[37], Solar [38], [39], Hydroelectric [40], [41], Biofuels [5], [18], [28]–[31], [42], Geothermal [43], Natural Gas and Petroleum drilling [44], [45], and Wind [22], [46]–[51].

### Where energy development occurs

The goal of this phase of the analysis was to partition the total area of new energy development among geographic regions. We ignored energy production techniques that had no significant cumulative areal impact as calculated above (i.e., end-use power generation, energy efficiency gains). For our regionalization analysis, we chose definitions of geographic regions that have maximal relevance to biodiversity yet are coarse-scaled enough to average over errors and uncertainty in more fine-scaled input data on energy resource availability and demand. For terrestrial impacts we used the 11 major habitat types of the United States, as defined by Nature Conservancy ecoregions [52]–[54]. For each major habitat type, we estimate the total area of new energy development, without attempting to specify where within each major habitat type development will take place. Within each major habitat type, there are a variety of land-uses, from relatively wild places to agricultural and urban systems. Thus specific siting decisions, while outside the scope of our analysis, will be important in determining actual biodiversity impact.

Throughout our analysis, we excluded certain areas as being protected or restricted from development, modeling our decision rules on those used in the Department of Energy's report “20% Wind Energy by 2030 [47].” We excluded areas that were protected areas with a Gap Analysis Program code of 1 or 2 (i.e., permanent protection excluding development), based on the Protected Area Database of the United States, version 4[55]. We also excluded airports, urban areas, and wetlands/water bodies from development, based on vector layers included with Environmental Systems Research Institute's (ESRI) ArcGIS package. Areas with an average slope greater than 20% were also excluded, based on a surface analysis of the GTOPO global digital elevation model [56]. Finally, for wind power we assumed that areas within 3 km of an airfield or urban area were not developable.

For each energy production technique, we partitioned its land use among regions in one of two methods. For some energy production techniques, continuous (i.e., interval or ratio scale) estimates of the supply of that resource were available for different geographic regions (Table S3 and Supplementary Text S1). For example, the Department of Energy publishes a continuous estimate of the water power potential in MW of the different hydrologic regions of the United States [57]. In these cases with continuous estimates of resource supply, we assumed that the area of energy development in each geographic region was proportional to the total supply in that region. For some resources, the geographic units in which data was available did not match those of our analysis units, and we used geographic information system (GIS) analyses to partition the resource among habitat types, making the simplifying assumption that the resource was evenly distributed within the original geographical units of the data. To give an example from one particularly dataset, potential biomass estimates were available from the National Renewable Energy Laboratories (NREL), summarized per county [58]. We calculated tonnes/km^2^ for each county, digitized the data to a 1 km raster resolution of the United States, and then used ESRI ArcGIS ZonalStatistics commands to sum up the total available biomass in each of our major habitat types.

Other energy production technologies had data on the supply of the resource that were categorical (i.e., ordinal scale). For example, NREL wind power maps rank sites on a scale of 1 to 7, based on the quantity of wind available as well as its consistency. In these cases with categorical data, we reclassified the U.S. into Excellent, Good, and Poor regions for development of that energy resource. In some cases a continuous estimate of a proxy for a resource was available rather than a direct estimate of power availability, and in these cases we classified the resource into categorical categories based on published opinion about what sites were developable. While our decision rules are admittedly arbitrary, they are derived from common GIS analysis for site selection in the energy industry, and we believe any reasonable set of decision rules would provide qualitatively similar results. In general, we looked at both the supply of a particular resource (e.g., how much sunlight is there?) and the demand (e.g., how far away is the nearest electric transmission line to carry the power to market?). The specific criteria we use are listed in Table S3. We then calculated how much of each geographic region was in the three categories (Excellent, Good, and Poor) using ESRI ArcGIS ZonalStatistics commands. Next, we assumed that the area of energy development in each geographic region was proportional to the area classified as Excellent in that region. If all areas categorized as Excellent were developed without meeting the total areal target, the remaining development was assumed to “spill-over” to the Good category, where it was similarly divided among geographic regions.

## Supporting Information

Table S1 Land-use intensity of production for new electricity generation capacity.
(0.04 MB DOC)

Table S2 Land-use intensity for new biofuel production.
(0.04 MB DOC)

Table S3 Decision criteria for classifying land.
(0.05 MB DOC)

Text S1 Technical Citations for Tables S1-S3
(0.03 MB DOC)

Supplementary Data S1 Detailed data on total U.S. area impacted (km^2^) by each energy sector
(0.02 MB XLS)

Supplementary Data S2 Detailed data on the area (km^2^) of U.S. habitat types affected by each energy sector
(0.04 MB XLS)
