# Supplementary material for: Energy Sprawl or Energy Efficiency: Climate Policy Impacts on Natural Habitat for the United States of America
Source: PLoS One. 2009 Aug 26;4(8):e6802. doi: 10.1371/journal.pone.0006802 (PMC2728545; doi:10.1371/journal.pone.0006802)
Supplement: Table S1 — Land-use intensity of production for new electricity generation capacity. (0.04 MB DOC) [file pone.0006802.s001.doc]

**Table S1. Land-use intensity of production for new electricity generation capacity.**

| **Type** | **Nameplate capacity factor (%)** | **Most compact km^2^/GW** | **Least compact km^2^/GW** | **Notes** | **Type of impact** |
| --- | --- | --- | --- | --- | --- |
| Hydropower | 44% | 62.2 | 354.8 | Capacity factor based on EIA Reference scenario, 2030 value. Based on 95% confidence interval of average of 25 randomly selected FERC dams. | Area submerged by lake |
| Geothermal | 85% | 7.5 | 103.6 | Capacity factor based on EIA Reference scenario, 2030 value. Most-compact estimate based on MIT’s Future of Geothermal [1], least-compact estimate on The Geysers plant in CA, which covers 30 mi^2^ and generates 750 MW. | Area covered by plant and access infrastructure, fragmented habitat |
| Solar Thermal | 29% | 25.9 | 51.8 | Capacity factor based on EIA Reference scenario, 2030 value. Based on DOE and NREL reports [2,3] | Area covered by plant and access infrastructure, fragmented habitat |
| Solar Photovoltaic (PV) | 28% | 51.8 | 129.5 | Capacity factor based on EIA Reference scenario, 2030 value. Based on DOE and NREL reports [2,3] | Area covered by plant and access infrastructure, fragmented habitat |
| Onshore Wind | 35% | 199.4 | 242.8 | Capacity factor based on EIA Reference scenario, 2030 value. Used AWE estimate as least compact estimate (http://www.awea.org/faq/wwt_environment.html) and DOE estimate [4] as most compact estimate. | Area covered by turbine and access infrastructure, fragmented habitat |
| Biomass for electricity- commercial generation | 75% | 2844 | 4294 | Capacity factor based on EIA Reference scenario, 2030 value. Least-compact estimate based on Keolian and Volk [5] for willow gasification, most-compact bound based on their most optimistic scenario of willow yield. | Area from which biomass is drawn to feed plant. |
| Nuclear | 91% | 3.02 | 4.78 | Capacity factor based on EIA Reference scenario, 2030 value. Values for plant area, mining requirements per KW-hr, and waste storage requirements per KW-hr are based on Spitzley, and Keoleian [6]. | Area covered by plant, as well as area for uranium mining and waste storage |
| End-use electricity (solar PV, geothermal, or wind) | NA | 0 | 0 | Limited land-use impact [7] | Infrastructure on already developed sites |
| Efficiency Gains | NA | 0 | 0 | Limited land-use impact [7] | Efficiency gains through changes inside existing building or infrastructure |

For each electric generation technology, we show the nameplate capacity factor, the percent of the stated generation capacity of the plant that is consistently available, as well as most-compact and least-compact estimates of the area requirements of new energy generation (km^2^/GW of nameplate capacity).
