# Supplementary material for: Energy Sprawl or Energy Efficiency: Climate Policy Impacts on Natural Habitat for the United States of America
Source: PLoS One. 2009 Aug 26;4(8):e6802. doi: 10.1371/journal.pone.0006802 (PMC2728545; doi:10.1371/journal.pone.0006802)
Supplement: Table S2 — Land-use intensity for new biofuel production. (0.04 MB DOC) [file pone.0006802.s002.doc]

**Table S2. Land-use intensity for new biofuel production.**

| **Type** | **Prop. of crop value embodied in biofuel** | **Most Compact m^2^/L** | **Least Compact m^2^/L** | **Notes** |
| --- | --- | --- | --- | --- |
| Domestic- Corn Ethanol | 0.76 | 2.5 | 2.9 | Least-compact estimate based on 2007 yields, a conversion rate of 2.7 gal/bu (0.29 L/L, or 0.40 L/kg), and the fact that 10% of planted land is not harvested. Most-compact estimate based on yield increases of 2 bu/year (70.4 L/yr or 50.8 kg/yr), starting with 151 bu/acre (1.31 L/m^2^ or 0.948 kg/m^2^) in 2007. Coproduct value from Wang [8]. |
| Domestic- Cellulosic Ethanol | 1.00 | 0.8 | 4.6 | Least compact estimate based on current switchgrass yields of 3.1 tons/acre (0.70 kg/m^2^) [9], and current conversion rates of 74.5 gals/ton (0.311 L/kg). Most compact estimate based on optimistic *miscanthus* yields of 13.8 tons per acre (3.09 kg/m^2^) [10], and "mature technology" conversion rates of 105.3 gal/ton (0.440 L/kg) [11]. Coproduct value based on current lack of market [12]. |
| Domestic- Ethanol from other feedstocks | 0.98 | 1.4 | 2.1 | Assumed sugarcane, used current production values from Brazil (see below). Coproduct value is surplus electricity, value estimated from cane yield in [13], assuming a rack price of ethanol of $1.80/gal ($0.47/L) and a U.S. electricity price of $0.0981/kWhr. |
| International- Imported Ethanol | 0.97 | 1.4 | 2.1 | Least-compact estimate based on current yields from FAO for Brazil, times 5/6 because sugarcane is harvested 5 of 6 years, times current conversion rates [13]. Most-compact estimate based on Projected yield and conversion increases from Macedo et al [13]. Coproduct value is surplus electricity, value estimated from cane yield in [13], assuming a rack price of ethanol of $1.80/gal ($0.47/L) and a Brazilian electricity price of $0.153/kWhr. |
| Domestic- Biodiesel | 0.46 | 16.0 | 20.2 | Least-compact estimate based on FAO yield data and conversion value from Gibbs et al. [14]. Most-compact estimate assumes that 0.5 bushel per acre per year (43 L/ha/yr) increase in soy production will continue. Coproduct value based on Huo et al. [15]. |
| International- Imported Biodiesel | 0.46 | 16.0 | 20.2 | Assumed same as domestic biodiesel. |
| Domestic- Liquids from Biomass | 0.83 | 1.0 | 1.4 | From 80.1 gals/dry ton (0.335 L/kg) and 110.9 gal/ton (0.463 L /kg) in NREL report [16] and 22 Mg/ha value of Heaton et al. [10]. Coproduct value inferred from alcohol price and yield relative to ethanol price and yield in Phillips et al. [16]. |

For each type of biofuel and its feedstocks, we show the proportion of the economic value of the crop that is used for biofuel production rather than other co-products, as well as most-compact and least-compact estimates of the area requirements of new energy generation, m^2^ of crop divided per liter. For all biofuels, the type of impact is defined as the area of new crop required as biofuel feedstock.
