# Supplementary material for: Energy Sprawl or Energy Efficiency: Climate Policy Impacts on Natural Habitat for the United States of America
Source: PLoS One. 2009 Aug 26;4(8):e6802. doi: 10.1371/journal.pone.0006802 (PMC2728545; doi:10.1371/journal.pone.0006802)
Supplement: Table S3 — Decision criteria for classifying land. (0.05 MB DOC) [file pone.0006802.s003.doc]

**Table S3.** Decision criteria for classifying land.

| **Energy technology** | **Supply** | **Demand** | **Data Sources** |
| --- | --- | --- | --- |
| Coal | Within coal basin (Excellent, Good, or Poor) | Distance to nearest navigable river or coal-bearing railroad (Excellent <25 km, Good 25-100 km, Poor > 100 km) | 2008 Carbon Sequestration Atlas (coal basins); Ventyx vector data (rivers and railroads). |
| CCS | Within known coal basin, oil or gas reservoir, or deep saline formation (Excellent, Good, or Poor) | Distance to nearest large (> 100 MW) coal or natural gas plant (Excellent < 25 km, Good 25-100 km, Poor > 100 km) | 2008 Carbon Sequestration Atlas (coal, oil, natural gas, and saline formations); Ventyx vector data (power plants) |
| Wind- onshore | Wind power class of NREL (Excellent 6-7, Good 4-5, Poor 3) | Distance to nearest power transmission line (Excellent < 25 km, Good 25-100 km, Poor > 100 km) | 50m NREL wind maps, where available, otherwise other sources of high-resolution maps for IA, MN, NY, WI, or TX, otherwise low-resolution NREL wind map; Ventyx vector data (transmission lines) |
| Wind- offshore | Wind power class of NREL (Excellent 6-7, Good 4-5, Poor 3) for sites in less than 30 m deep water | Distance to nearest power transmission line (Excellent < 25 km, Good 25-100 km, Poor > 100 km) | 50m NREL wind maps, where available, otherwise other sources of high-resolution maps for IA, MN, NY, WI, or TX, otherwise low-resolution NREL wind map, otherwise NASA’s Prediction of Worldwide Energy Resource project Surface meteorology and Solar Energy dataset; Ventyx vector data (transmission lines); GTOPO1 (bathymetry) |
| Geothermal | U.S. geothermal resources map (Excellent > 200 ºC, Good 150-200 ºC, Poor < 150 ºC), Alaska map of geothermal resources (if delineated as exploitable, Good) | Distance to nearest power transmission line (Excellent < 25 km, Good 25-100 km, Poor > 100 km) | U.S. Geothermal Resources Map, otherwise Alaska assessment of geothermal resources; Ventyx vector data (transmission lines) |
| Hydropower | Continuous measurement of total MW potential by HUC regions | Distance to nearest power transmission line less than 25 km, else unsuitable | DOE’s Water Energy Resources of the United States; Ventyx vector data (transmission lines) |
| Solar PV | Annual kilowatt hours for optimally tilted surface (Excellent > 6000, Good 4500-6000, Poor 3000-4500) | Distance to nearest power transmission line (Excellent < 25km, Good 25-100km, Poor > 100 km) | NREL estimates for the lower 48 states, otherwise NASA’s Prediction of Worldwide Energy Resource project Surface meteorology and Solar Energy dataset; Ventyx vector data (transmission lines) |
| Solar Thermal | Annual kilowatt hours for direct normal insolation (Excellent > 6000, Good 4500-6000, Poor 3000-4500) | Distance to nearest power transmission line (Excellent < 25km, Good 25-100km, Poor > 100 km) | NREL estimates for the lower 48 states, otherwise NASA’s Prediction of Worldwide Energy Resource project Surface meteorology and Solar Energy dataset; Ventyx vector data (transmission lines) |
| Nuclear | Near major water supplies for cooling (Excellent, Good, or Poor); Acceptable earthquake risk (less than a 2% chance of an earthquake with peak horizontal acceleration of 0.8 G within 50 years). | Distance to nearest navigable river or coal-bearing railroad (Excellent <25 km, Good 25-100 km, Poor > 100 km); Distance to nearest power transmission line (Excellent < 25 km, Good 25-100 km, Poor > 100 km) | GTOPO1 (bathymetry of oceans and Great Lakes); U.S. National Water Polygon Feature Areas, ESRI data (location of interior water bodies, non-permanent features like swamps excluded); United States National Seismic Hazard Map; Ventyx vector data (rivers, railroads, and transmission lines) |
| Natural Gas- domestic | Within known natural gas basin (Excellent, Good, or Poor) | Distance to nearest natural gas line (Excellent < 25 km, Good 25-100 km, Poor > 100 km) | Ventyx vector data (natural gas lines and oil basins) |
| Natural Gas- international | Continuous measurement of billion cubic feet of gas remaining within natural gas basins | None | World Petroleum Assessment 2000 [17] |
| Oil- domestic, onshore lower 48 | Within known oil producing region (Excellent, Good, or Poor) | Distance to nearest oil refinery (Excellent < 100 km, Good 100-200 km, Poor > 200 km) | 1995 National Oil and Gas Assessment; Ventyx vector data (oil refinery locations) |
| Oil- domestic, offshore lower 48 | Within known oil producing basin (Excellent, Good, or Poor); Water Depth (Excellent < 200 m, Good 200-3000 m, Poor > 3000 m) | Within Exclusive Economic Zone (Excellent, Good, or Poor) | World Petroleum Assessment 2000; Ventyx vector data (current oil platforms); Mann et al. map of known large oil basins [18]; GTOPO1 (bathymetry of oceans); ESRI vector files of U.S., Canada, and Mexico (calculation of EEZ). |
| Oil- international, onshore | Continuous measurement of million barrels of oils remaining within oil basins | None | World Petroleum Assessment 2000 [17] |
| Oil- international, offshore | Continuous measurement of million barrels of oils remaining within oil basins | None | World Petroleum Assessment 2000 [17] |
| Biofuels- domestic corn ethanol | GAEZ suitability class for growing corn (Excellent 1-2, Good 3, Poor 4-7) | Distance to nearest biofuel refinery, current or proposed (Excellent < 100 km, Good 100-200 km, Poor > 200 km) | GAEZ suitability data; Ventyx vector data (biofuel refineries) |
| Biofuels- domestic soy biodiesel | GAEZ suitability class for growing soy (Excellent 1-2, Good 3, Poor 4-7) | Distance to nearest biofuel refinery, current or proposed (Excellent < 100 km, Good 100-200 km, Poor > 200 km) | GAEZ suitability data; Ventyx vector data (biofuel refineries) |
| Biofuels- domestic cellosic ethanol | GAEZ suitability class for any crop (Excellent 1-2, Good 3, Poor 4-7) | None | GAEZ suitability data; |
| Biofuels- international ethanol | GAEZ suitability class for sugarcane (Excellent 1-2, Good 3, Poor 4-7) | None | GAEZ suitability data; |

Land was classified as Excellent, Good, or Poor for the development of particular energy technologies. All other areas considered unsuitable. Within the United States, all areas that are permanently excluded (see text) from development are classified as unsuitable. Both supply and demand/market access issues were considered for each technology. If a particular place was classified in one category with regard to supply issues and another category with regard to demand/market access issues, the place is assigned to the lowest category. For example, a site with Excellent geothermal resources but 50km from a power line is classified as Good.
