# Supplementary material for: Energy Sprawl or Energy Efficiency: Climate Policy Impacts on Natural Habitat for the United States of America
Source: PLoS One. 2009 Aug 26;4(8):e6802. doi: 10.1371/journal.pone.0006802 (PMC2728545; doi:10.1371/journal.pone.0006802)
Supplement: Text S1 — Technical Citations for Tables S1-S3 (0.03 MB DOC) [file pone.0006802.s004.doc]

**Works Cited**

1. MIT (2006) The future of geothermal energy: Impact of Enhanced Geothermal Systems (EGS) on the United States in the 21st century. Cambridge, MA: Massachusetts Institute of Technology.

2. Tsoutsos T, Frantzeskaki N, Gekas V (2005) Environmental impacts from the solar energy technologies. Energy Policy 33: 289-296.

3. Denholm P, Margolis RM (2008) Impacts of Array Configuration on Land-Use Requirements for Large-Scale Photovoltaic Deployment in the United States. Washington, DC: Department of Energy.

4. DOE (2008) 20% wind energy by 2030. Oak Ridge, TN: Department of Energy, Office of Scientific and Technical Information.

5. Keoleian GA, Volk TA (2005) Renewable energy from willow biomass crops: Life cycle energy, environmental, and economic performance. Critical Reviews in Plant Sciences 24: 385-406.

6. Spitzley DV, Keoleian GA (2004) Life cycle environmental and economic assessment of willow biomass electricity: A comparison with other renewable and non-renewable sources. Ann Arbor, MI: Center for Sustainable Systems, University of Michigan.

7. Dincer I, Rosen MA (1999) Energy, environment, and sustainable development. Applied Energy 64: 427-440.

8. Wang M. Updated energy and greenhouse gas emission results of fuel ethanol; 2005; San Diego, CA. Available at http://www.transportation.anl.gov/pdfs/TA/375.pdf.

9. Schmer MR, Vogel KP, Mitchell RB, Perrin RK (2008) Net energy of cellulosic ethanol from switchgrass. Proceedings of the National Academy of Sciences of the United States of America 105: 464-469.

10. Heaton E, Voigt T, Long SP (2004) A quantitative review comparing the yields of two candidate C4 perennial biomass crops in relation to nitrogen, temperature, and water. Biomass and Bioenergy 27: 21-30.

11. Lynd LR, Laser MS, Bransby D, Dale BE, Davison B, et al. (2008) How biotech can transform biofuels. Nature Biotechnology 26: 169-172.

12. Fargione J, Hill J, Tilman D, Polasky S, Hawthorne P (2008) Land clearing and the biofuel carbon debt. Science 319: 1235-1238.

13. Macedo IC, Seabra JEA, Silva JEAR (2008) Green house gases emissions in the production and use of ethanol from sugarcane in Brazil: The 2005/2006 averages and a prediction for 2020. Biomass and Bioenergy 32: 582-595.

14. Gibbs HK, Johnston M, Foley JA, Holloway T, Monfreda C, et al. (2008) Carbon payback times for crop-based biofuel expansion in the tropics: the effects of changing yield and technology. Environmental Research Letters 3.

15. Huo H, Wang M, Bloyd C, Putsche V (2008) Life-cycle assessment of energy and greenhouse gas effects of soybean-derived biodiesel and renewable fuels. Chicago: Center for Transportation Research, Argonne National Laboratory.

16. Phillips S, Aden A, Jechura J, Dayton D, Eggeman T (2007) Thermochemical ethanol via indirect gasification and mixed alcohol synthesis of lignocellulosic biomass. Washington, DC: U.S. Department of Energy.

17. USGS (2000) World Petroleum Assessment. Washington, DC: United States Geological Survey.

18. Mann P, Horn M, Cross I (2007) Major World Oil and Gas Fields. Austin, TX: The University of Texas.
